# Improving the Through-Thickness Thermal Conductivity of Carbon Fiber/Epoxy Laminates by Direct Growth of SiC/Graphene Heterostructures on Carbon Fibers

**DOI:** 10.1021/acsomega.3c01951

**Published:** 2023-06-29

**Authors:** Anastasios Karakassides, Abhijit Ganguly, Constantinos E. Salmas, Preetam K. Sharma, Pagona Papakonstantinou

**Affiliations:** † School of Engineering, Ulster University, Belfast BT15 1AP, Northern Ireland, U.K.; ‡ Department of Materials Science & Engineering, University of Ioannina, 45110 Ioannina, Greece

## Abstract

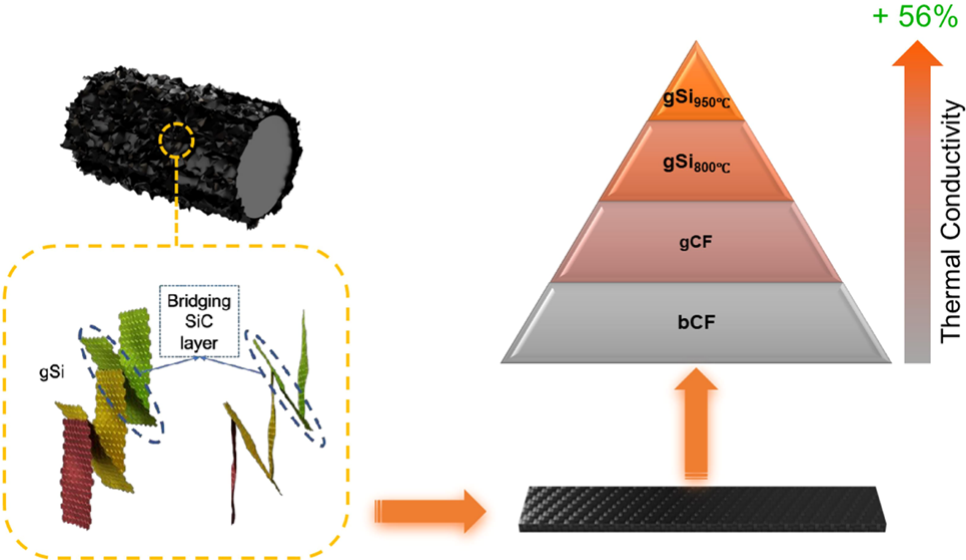

Poor thermal conductivity in the through-thickness direction is a critical limitation in the performance of carbon fiber-reinforced polymer (CFRP) composites over a broad range of applications in the aviation industry, where heat dissipation is required (e.g., battery packs, electronic housing, and heat spreaders). In this work, it is demonstrated for the first time that a hierarchical network of vertically oriented graphene nanoflakes (GNFs), with nanoconfined silicon carbide (SiC) nanocrystals, self-assembled on carbon fibers (CFs) can provide significant improvement to the thermal conductivity (TC) of CFRPs in the through-thickness direction. The vertically aligned SiC/GNF heterostructures were grown directly on CFs for the first time by single-step plasma-enhanced chemical vapor deposition (PECVD) employing tetramethylsilane (TMS) and methane (CH_4_) gases at temperatures of 800 and 950 °C. At the deposition temperature of 950 °C, the controlled introduction of SiC/GNF heterostructures induced a 56% improvement in through-thickness TC over the bare CFRP counterparts while simultaneously preserving the tensile strength. The increase in thermal conductivity is accomplished by SiC nanocrystals, which serve as linkage thermal conducting paths between the vertical graphene layers, further enhancing the smooth transmission of phonons in the vertical direction. The work demonstrates for the first time the unique potential of novel SiC/GNF heterostructures for attaining strong and thermally conductive multifunctional CFRPs.

## 1 Introduction

In the aircraft industry, there is a drive toward the concept of hybrid electric-powered or more-electric aircraft (MEA) and eventually of an all-electric aircraft.^1−3^ This increased tendency for the amplified usage of electrical power on aircraft emanates from the need to enhance the overall aircraft efficiency, decrease fuel consumption, and thus reduce emissions of greenhouse gases.^4,5^ Coupled with this need, there is a rising trend for the use of carbon fiber-reinforced polymer (CFRP) composites due to their superior mechanical characteristics and lightweight nature.^6^

The development of MEA has brought about an intensive integration and miniaturization of onboard high-power electronic and multifunctional devices, which create the additional requirement to develop lightweight electronic packing composite materials with superior thermal conductivity (TC) to channel away the rising heat produced from the electronic components.^7^ If the heat generated is not removed, the internal temperature of the device components will continue to increase, putting at risk the system reliability and efficiency.^8^ As a result, superior TC in CFRPs becomes imperative, being capable of removing heat from electronic devices.

Because of the poor heat dissipation ability of the epoxy matrix, the through-thickness TC of CFRP (<1 W/mK) seriously limits its application in thermal management systems (e.g., composite battery enclosures, electronic housing, etc.). It is well known that polymeric materials present poor TCs varying from 0.1 to 0.5 W/(mK),^9^ credited to the irregular molecule chains’ random rotation and vibration, causing reduced phonon group velocities and decreased phonon mean free paths. A popular approach to improve the TC of polymer matrices is to incorporate thermally conductive nanofillers based on ceramic (e.g., SiC,^10−13^ Al_2_O_3_, BN), metallic (e.g., Cu, Al), and carbon nanomaterials (e.g., graphene, carbon black, carbon nanotubes (CNTs)).^14−16^ However, in order to obtain an appreciable improvement in TC (up to 15 W/(mK)), high filler loadings are required (50–70 wt % filler content),^17,18^ which usually result in the loss of processability and a concomitant degradation of mechanical properties.^19−21^ To date, there is an enduring challenge to boost the TC of polymer composites by simultaneously engineering the molecular interactions of filler/filler and filler/polymer interfaces, which are vital for achieving efficient isotropic thermal transport.

Among the filler materials, graphene has attracted a great deal of attention because of its excellent intrinsic thermal conductivity (>4000 W/(mK))^22^ along the basal plane. However, randomly distributed graphene layers in the matrix show limited improvement due to insufficient interconnected paths between the graphene sheets and weak thermal coupling among the graphene/polymer matrix and graphene/graphene interfaces. Therefore, one would hypothesize that the effective alignment of graphene layers in the vertical orientation within the matrix, with strong interlayer coupling between the layers, so that the intrinsic thermal properties of graphene along the basal plane can be fully utilized, would improve the through-thickness TC of the composite.

Experimental and theoretical studies have revealed that the nature of the interfacial bonding between layers is a significant factor for phonon transport and hence TC in the through-plane direction. Generally, the weak van der Waals (vdW) forces in layered materials hinder phonon transport^23^ when compared to covalent or ionic forces. In line with the above, TC in layered crystals, where layers are bonded by weak van del Waals forces, is considerably inferior in the through-plane direction (*K*_⊥_) compared to the in-plane direction, *K*_//_ (e.g., *K*_//_ ≈ 2200 W/mK vs *K*_⊥_ ≈ 6 W/mK for graphite).^22^

Lately, manipulation of interlayer coupling between two-dimensional materials that constitute vdW heterojunctions has become a promising approach to enhance thermal transport.^24^ It has been found that interlayer rotation,^25^ application of compressive strains,^26^ defect concentration, and number of layers^27^ can influence the thermal transport characteristics in the in-plane and out-of-plane directions in vdW heterostructures.^25^

Recently, we synthesized self-assembled vertically oriented graphene nanoflakes (GNFs) directly onto carbon fibers (CFs) by means of plasma-enhanced chemical vapor deposition (PECVD) and successfully demonstrated that they constitute a promising reinforcement nanointerface for enhancing the fracture toughness of CFRP without deteriorating the in-plane mechanical properties.^28,29^ One of the great assets of GNFs is that they can be grown without the need for a metal catalyst, demonstrating a substantial cost reduction for large-scale manufacturing, when compared to other carbon nanoreinforcements like oriented CNTs. Although the through-volume electrical conductivity of the GNF composites improved more than 5 times compared to the pristine CFRP counterparts, the thermal properties of the novel hybrid laminate remain unexplored.

In this work, we measure the thermal transport properties of CFRPs with new three-dimensional (3D) silicon carbide (SIC)/graphene hybrid nanostructures directly grown on CFs. The hybrid nanostructures comprise vertical graphene nanoflakes and SiC nanocrystals confined between the graphene layers. By controlling the deposition conditions, we manipulate the synthesized 3D hybrid architectures and tune their thermal transport performance. Generally, SiC crystals exhibit high thermal conductivity (∼490 W/mK) and hence are used as thermal conductivity fillers.^30^ As mentioned previously, much effort has been dedicated over the last few years to enhancing the thermal performance of CFRPs via modification of the epoxy matrices utilizing mixtures of graphene/graphitic nanoplatelets and SiC crystals at different weight fractions. However, to date, there is no related research work reporting the direct growth of a graphene/SiC heterostructure on CF in the literature with a view to enhancing the thermal conductivity in the through-thickness direction in laminates.

In this work, in an effort to address the challenge of improving the thermal conductivity of CFRPs, we report a first-of-its-kind study on the direct growth of graphene nanoflakes (GNFs) intercalated with SiC nanocrystals. To elucidate the role of the SiC/graphene heterostructure on the mechanical and thermal conductivity characteristics of laminates, we conducted a systematic investigation of the structure–property (mechanical and thermal conductivity) relationships and their comparison with bare carbon fiber (CF) and graphene nanoflake-coated CF (CF/GNF) counterparts (which acted as reference samples). Scanning electron microscopy (SEM), Raman, and mechanical characterization data for CF and CF/GNF counterparts were reproduced from our previous work^29^ and are presented in the Supporting Information. Such a comparison is important to understand the role of SiC incorporation in the GNFs. The results indicated that the thermal conductivity of the fabricated CFRP can be enhanced dramatically by the incorporation of SiC nanocrystals onto the grown GNFs, without actually deteriorating their in-plane tensile strength. The outcomes of this article revealed the unique capability of SiC/GNFs heterostructures, as exceptional nanoreinforcements, as well as thermally conducting interfaces, for realizing at the same time strong and thermally conductive multifunctional CFRPs. By simultaneously harnessing the strong phonon transport along the graphene layers and SiC nanocrystals and the covalent interactions between them, we obtained a 56% enhancement in through-plane thermal conductivity compared to bare CFRP composites.

## 2 Materials and Methods

Herein, CF plain weaves (Pyrofil TR30S 3k), possessing a mass per unit area of 210 g m^–2^, were supplied from Easy Composites Ltd. (Easy Composites Ltd., U.K.). The matrix selected for the manufacture of the laminated structures was the IN2 epoxy resin, purchased again from Easy Composites Ltd. Argon (Ar) along with methane (CH_4_) used for the depositions was bought from BOC Ltd. (BOC Ltd., U.K.). Tetramethylsilane (TMS: Si(CH_3_)_4_) was purchased from Sigma-Aldrich (Sigma-Aldrich, U.S.).

### 2.1 Direct Growth of SiC/GNFs onto CFs by One-Step rf-PECVD

The deposition of SiC/GNFs was accomplished using an rf-PECVD system (Zhengzhou Protech Technology Co., Ltd.), with Ar, CH_4_, and Si(CH_3_)_4_ (TMS) as precursors. The main advantage of TMS for producing SiC materials is that the Si–C bond already exists in the precursor molecule. The CF weaves were set onto a custom-built quartz boat holder of 25 cm total length, located in the mid-area of the furnace tube, with the assistance of a long metallic hook. When the background pressure inside the tube reached 8 × 10^–4^ Torr, Ar (30 sccm) was inserted to attain the selected pressure of 3 × 10^–2^ Torr. Then, the temperature was raised to 800 or 950 °C. Once the chosen temperature was achieved, a mix of TMS and CH_4_ (Table 1) was inserted in the tube furnace and SiC/GNF growth started, following the plasma striking at a power of 500 W, for a period of 30 min. Subsequent to the termination of SiC/GNF deposition, a continuous Ar stream (30 sccm) was utilized to reduce the temperature of the coated CFs.

**Table 1:** Growth Conditions of SiC/GNFs on CF^a^

sample	gCF	gSi_800°C_	gSi_950°C_
RF power (W)	500	500	500
temperature (°C)	800	800	950
time (min)	30	30	30
pressure (×10^–2^) (Torr)	3	3	3
TMS/CH_4_ (sccm)	0/20	8/2	8/2

a gCF denotes graphene nanoflakes on CF; gSi_800°C_ and gSi_900 C_ denote SiC/GNFs on CFs grown at 800 and 900 °C respectively.

### 2.2 Material Characterization

The surfaces of the deposited SiC/GNFs onto CFs, along with the fractured surface of the composites attained from the mechanical characterization, were inspected via scanning electron microscopy (FESEM, HITACHI SU5000). In order to examine in depth the heterostructure, apart from SEM, a transmission electron microscope (TEM, Jeol JEM-2100F) was used. Regarding the samples’ preparation, a suspension of SiC/GNFs in methanol was drop-cast onto the lacey carbon grid. X-ray diffraction (XRD, Panalytical Empyrean Series 3) was utilized to identify crystal orientations and possible phases (Supporting Information S1, Figure S1). X-ray photoelectron spectroscopy (XPS) (ThermoFisher Scientific-ESCALAB QXi X-ray) was utilized for elemental analysis and identification of bonding configurations on the samples’ surfaces. Raman spectroscopy was utilized for examining the SiC/GNFs’ electronic structure, along with identifying any differences on the surface of deposited weaves, by means of a Renishaw Invia Qontor system (532 nm excitation wavelength).

### 2.3 Vacuum-Assisted Resin Infusion (VARI) Procedure

All composite samples were fabricated through a vacuum-assisted resin infusion process (VARI) at 25 °C. Both the resin and the hardener were slowly mixed at a mass ratio of 10:3 as suggested by the manufacturer. The manufactured composites comprised 12 fabric layers, stacked in a piling arrangement of 0/90° ([0/90]_12_). The laminates were cured primarily at 25 °C for 24 h and subsequently underwent postcuring for 6 h at 60 °C. The manufactured composites possessed a thickness of 3 mm and were cut to suitable dimensions for all mechanical measurements under examination (*L*_M-I_ × *W*_M-I_ = 125 mm × 20 mm; *L*_M-II_ × *W*_M-II_ = 190 mm × 20 mm; *L*_Ten_ × *W*_Ten_ = 210 mm × 20 mm). For all mechanical tests, two SiC/GNF-deposited plain weaves were positioned at the mid-area of each specimen, facing each other, creating in this way a new reinforced interface. It should be noted that the void content of fabricated composites was assessed through image recognition techniques that can be found in the Supporting Information (Supporting Information S9, Figure S9), and it was found to be around 2.5%.

### 2.4 Mechanical Characterization

Modes I and II interlaminar fracture toughness and tensile strength tests were evaluated following protocols described in our earlier work.^29^

### 2.5 Thermal Conductivity Measurements

Thermal conductivity measurements were conducted on a TCi Thermal Conductivity Analyzer (C-Therm Technologies Ltd., Canada) using the modified transient plane source (MTPS) method at room temperature in accordance with ASTM D7984.^31^ Regarding the samples’ preparation, water was selected as a medium for achieving better contact between the sensor and the samples.

## 3 Results and Discussion

### 3.1 SEM and Raman Characterization

SEM images were used to evaluate the morphology of the deposited SiC/GNF nanostructure on CFs and therefore acquire information on the deposition conditions–nanostructure–performance triangular correlation. The SiC/GNF coating was restrained on the CFs fabrics’ upper surface because direct plasma exposure on the other side was hindered by the quartz boat, which was used as a sample holder.

**Figure 1:**
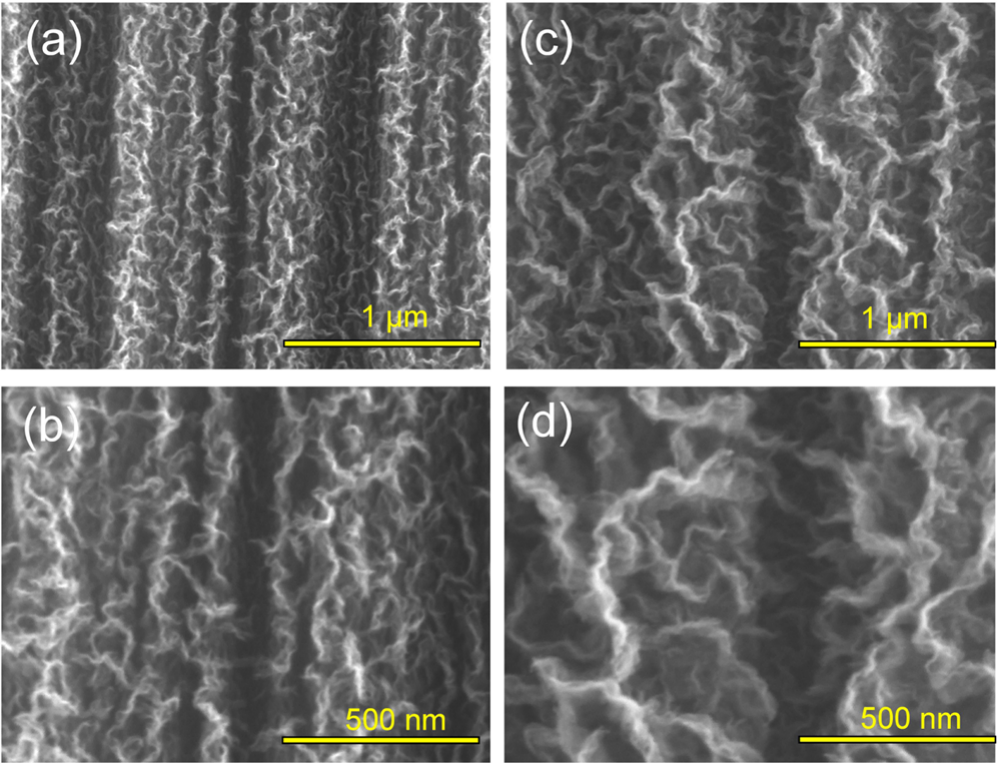
SEM micrographs of (a, b) SiC/GNFs on CF at 800 °C: gSi_800°C_ and (c, d) SiC/GNFs on CF at 950 °C: gSi_950°C_.

Figure 1 shows SEM images of SiC/GNFs deposited onto CFs under the chosen growth conditions. A comparison with the reference bare CF (bCF) and gCF samples, reproduced from our previous work,^29^ is provided in the Supporting Information, Figure S1. All samples presented a self-assembled web of perpendicularly oriented graphene sheets, creating a structure similar to that of a maze. The bCF (Figure S1a,b) had a diameter of ∼6.13 ± 0.49 μm, with apparent surface ridges derived from the CF fabrication process. Generally, no significant variations could be observed between the two SiC/GNF samples (gSi_800°C_ and gSi_950°C_, Figure 1a–d), with both exhibiting a corrugated morphology. The only perceivable difference was in the vertical length (height) of the corrugated nanosheets, with the high-temperature SiC/GNF sample (gSi_950°C_, Figure 1c,d) showing longer corrugated outgrowths (7.60 ± 0.36 μm) when compared to the lower-temperature one (gSi_800°C_, Figure 1a,b) (7.20 ± 0.41 μm). The longer outgrowths (gSi_950°C_) are mainly attributed to the higher dissociation rates of precursor gases, leading to a higher growth rate, at the elevated temperature of 950 °C. In contrast, gCF (6.80 ± 0.45 μm), which was fabricated at a higher CH_4_ flow rate and without TMS, demonstrated a less wavy appearance (Figure S1c,d).

**Figure 2:**
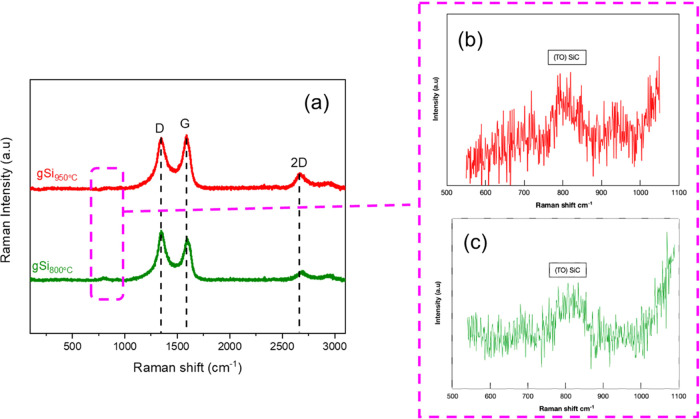
(a) Raman characterization of all samples. SiC/GNFs on CF at 800 °C: gSi_800°C_ and SiC/GNFs on CF at 950 °C: gSi_950°C_. (b, c) are Raman spectra of gSi_950°C_ and gSi_800°C_, respectively, of the dotted region indicated at (a).

Raman scattering was executed onto the deposited CF weaves in order to evaluate the existence of defects, the number of graphene layers (e.g., graphitic grade of the GNFs), and the presence of SiC phase. The obtained spectra (Figure 2a) included eminent vibrational modes, at 1345 cm^–1^ (D band), 1580 cm^–1^ (G band), and 2690 cm^–1^ (2D band). The small band around 800 cm^–1^ that (Figure 2b,c) was detected in both SiC/GNF samples is representative of the SIC transverse optical (TO) mode.^32^ Analysis of the spectra revealed that gSi_800°C_ exhibited an *I*_D_/*I*_G_ ratio of 1.08, whereas the gSi_950°C_ sample had a lower *I*_D_/*I*_G_ ratio of 1.01. A comparison with the reference bare CF (bCF) and gCF samples, reproduced from our previous work,^29^ is provided in the Supporting Information, Figure S2. The higher *I*_D_/*I*_G_ values for the SiC/GNFs with respect to pure GNFs (gCF had a ratio of 0.97) indicate a more defective structure, and this could be attributed to the introduction of Si (sp^3^-type bonding) in the graphene lattice. However, the drop in the *I*_D_/*I*_G_ ratio, when comparing gSi_800°C_ and gSi_950°C_ samples (1.08 to 1.01), can be rationalized bearing in mind the higher degree of graphitization expected for SiC/GNFs grown at elevated temperatures. All samples exhibited *I*_2D_/*I*_G_ values below 1, indicating the existence of multilayer graphene.^28^

### 3.2 TEM Analysis

**Figure 3:**
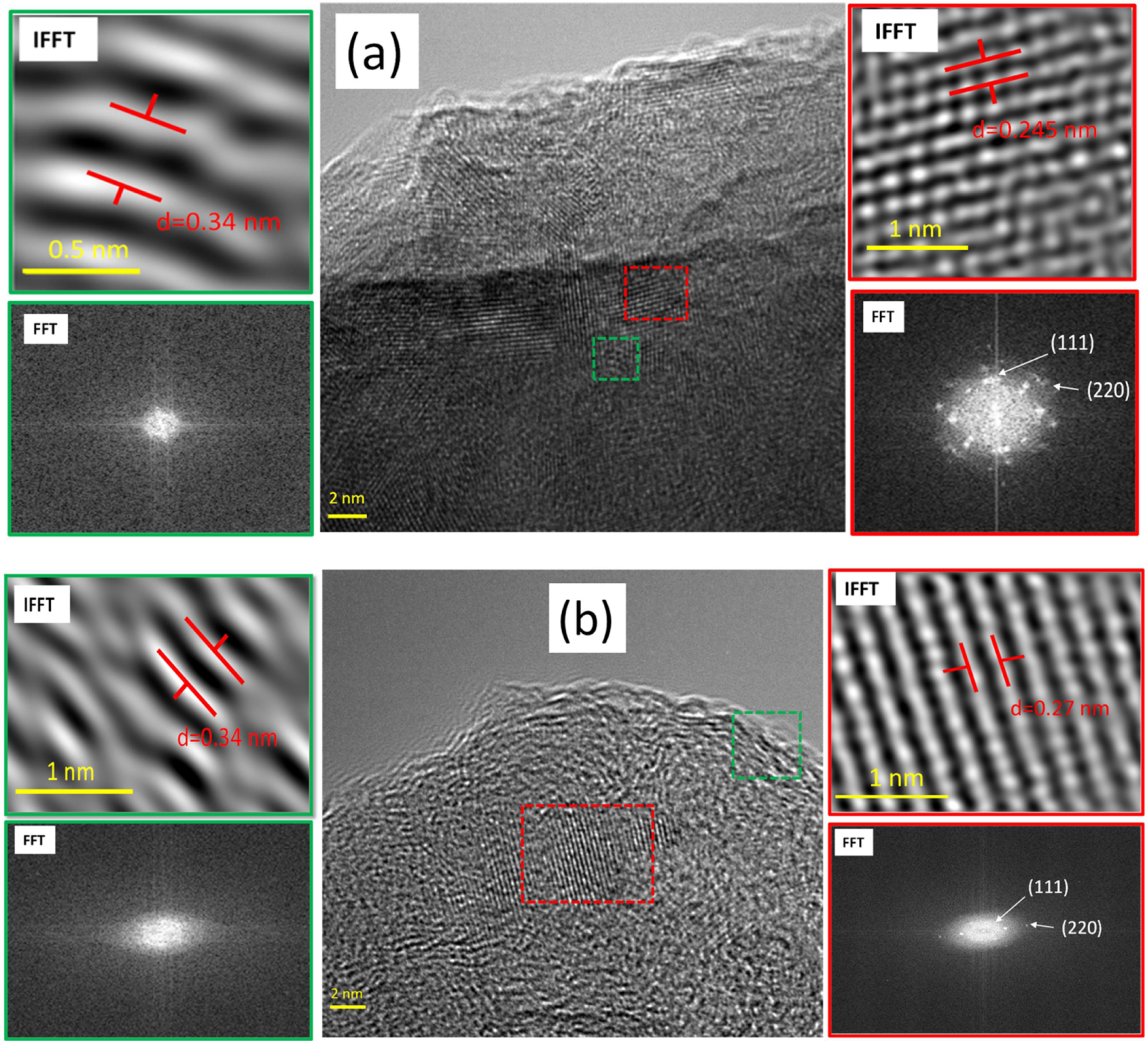
TEM analysis of (a) gSi_800°C_: SiC/GNFs on CF at 800 °C (top panels), and (b) gSi_950°C_: SiC/GNFs on CF at 950 °C (bottom panels). The green squared windows indicate the GNFs, and the red ones indicate the SiC. The term FFT represents the fast Fourier transform computing method, and the term IFFT represents the inverse fast Fourier transition method.

Figure 3 illustrates the high-resolution TEM analysis of gSi_800°C_ (Figure 3a) and gSi_950°C_ (Figure 3b) samples. The analysis revealed approx. 4–5 layers with an interlayer spacing of 0.34 nm for gSi_800°C_ and about 9–10 layers with an interlayer spacing of 0.34 nm for gSi_950°C_, which correspond to the (002) plane of graphitic carbon.^33−36^ Apart from these, SiC crystalline nanodomains of about 3–7 nm lateral size can be observed, which basically define the lateral size of the heterostructures. The lattice spacings measured from these nanodomains were 0.245 nm for gSi_800°C_ and 0.27 nm for gSi_950°C_, corresponding to the (111) and (220) planes of SiC^37,38^ (Figure 3a,b fast Fourier transform (FFT) images). The interlayer spacing of gSi_950°C_ (0.27 nm) is larger than the typical 0.245 nm spacing of SiC, which could be attributed to additional O atoms introduced between the layers of SiC, enlarging the spacing, as was confirmed by XPS analysis discussed below. It is expected that the lateral size of the SiC nanodomains would increase at growth temperatures larger than 950 °C, however, at the expense of the mechanical strength of carbon fibers.

It should be noted that gSi_950°C_ exhibited SiC lattice fringes of increased length (Figure 3b, green dotted window) when compared to the ones of gSi_800°C_ (Figure 3a, green dotted window). This could be attributed to the higher deposition temperature of the gSi_950°C_ sample, which led to an increased crystallization level of SiC crystals.

Considering the above TEM results, the SiC crystals, located between the graphene layers, are covalently bonded to graphene^39^ and serve to increase the interconnectivity between the graphene sheets.

### 3.3 XPS Analysis

**Figure 4:**
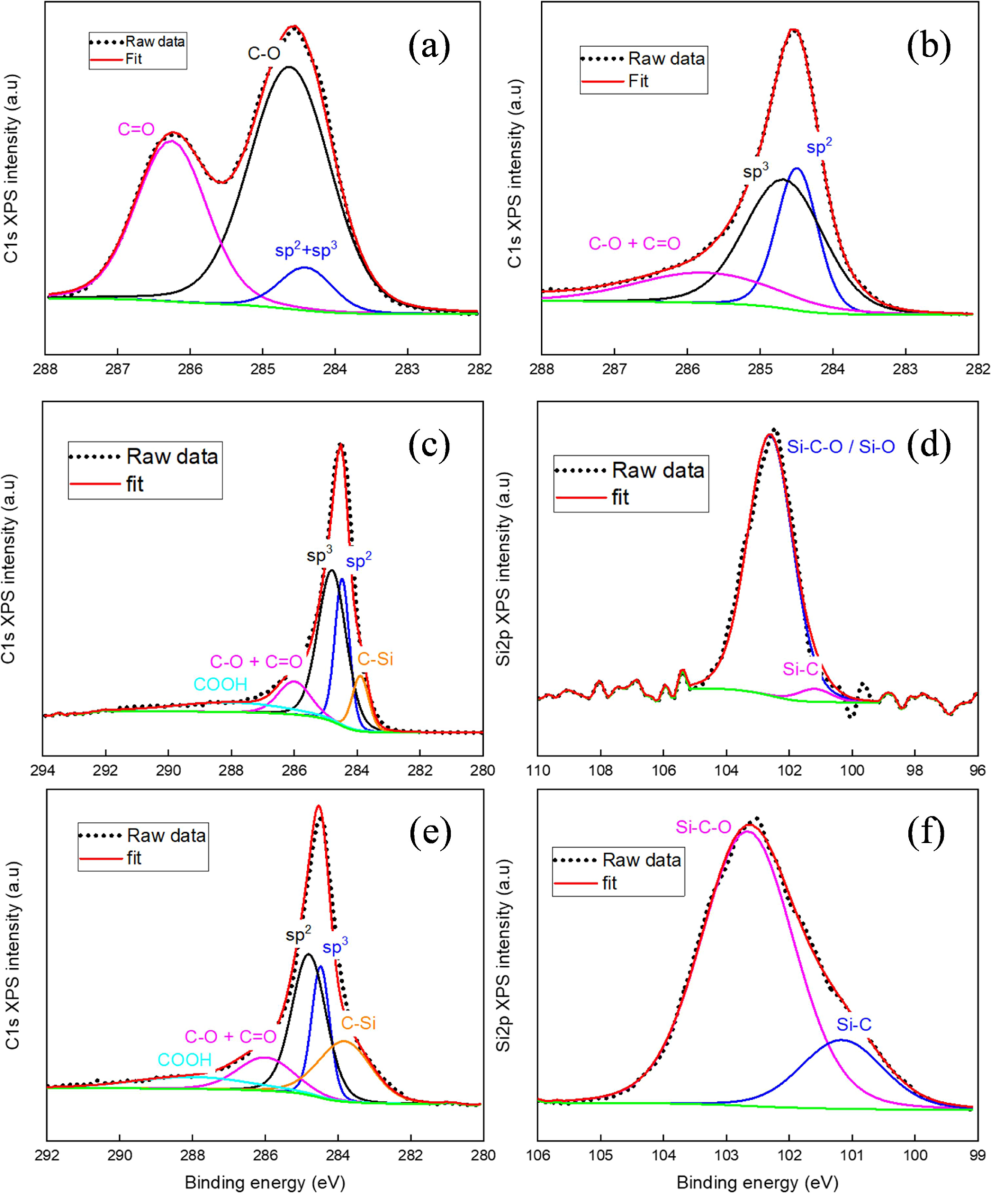
XPS analysis of bCF, gCF, gSi_800°C_, and gSi_950°C_ samples. (a) C 1s band of the bCF sample, (b) C 1s band of the gCF sample, (c) C 1s band of the gSi_800°C_ sample, (d) Si 2p band of the gSi_800°C_ sample, (e) C 1s band of the gSi_950°C_ sample, and (f) Si 2p band of the gSi_950°C_ sample.

XPS (Figure 4) revealed significant differences in carbon, oxygen, and Si atomic concentrations (Table 2) between the samples, suggesting possibly different interactions among the SiC/GNFs and the matrix. By increasing the temperature of the deposition from 800 to 950 °C, a tremendous increment in the Si content (from 1.7 to 21.5 atom %) was observed. In both samples (gSi_800°C_, gSi_950°C_), the C 1s core-level spectra were deconvoluted using the sp^2^, sp^3^, and C–O + C=O, COOH,^40^ and C–Si bonds (Table 3). The C–Si contents were estimated as 3 and 8.7% for gSi_800°C_ and gSi_950°C_ samples, respectively, which was in good agreement with the higher Si atomic percentage observed for gSi_950°C_. The sp^2^ atomic percentage decreased, while the sp^3^ atomic percentage increased, with temperature due to the higher Si incorporation into the sp^2^ network of GNFs during the growth. The main differences between gSi_800°C_ and gSi_950°C_ and the rest of the samples (bCF, gCF) could be identified as well from the Si 2p XPS spectra. From the deconvolution of the Si 2p spectra, Si–C bonds were clearly observed in gSi_800°C_ and gSi_950°C_ around 101.20 and 101.16 eV, respectively^41^ (Table 4), indicating the formation of SiC nanocrystals, confirming their observation from the TEM analysis.

**Table 2:** Atomic Concentrations of C, O, and Si of the Investigated Samples

sample	C 1s%	O 1s%	Si 2p%
bCF	76.15	23.85	
gCF	96.16	3.84	
gSi_800°C_	90.40	7.87	1.73
gSi_950°C_	47.62	30.89	21.49

**Table 3:** Atomic Concentrations of the Deconvoluted C 1s XPS Signal for All Samples

sample	peak	binding energy (eV)	concentration (%)
bCF	sp^2^ + sp^3^	284.40	15 ± 0.33
	C–O	284.72	51 ± 0.28
	C=O	286.26	34 ± 0.21
gCF	sp^2^	284.50	31 ± 0.23
	sp^3^	284.64	46 ± 0.35
	C–O + C=O	285.57	23 ± 0.19
gSi_800°C_	sp^2^	284.48	23 ± 0.13
	sp^3^	284.80	45 ± 0.26
	C–O + C=O	286.00	11 ± 0.21
	C–Si	283.90	10 ± 0.28
	COOH	288.00	11 ± 0.13
gSi_950°C_	sp^2^	284.48	18 ± 0.23
	sp^3^	284.80	35 ± 0.26
	C–O + C=O	286.00	14 ± 0.17
	C–Si	283.81	23 ± 0.16
	COOH	288.00	10 ± 0.15

**Table 4:** Atomic Concentrations of the Deconvoluted Si 2p XPS Signal for All Samples

sample	peak	binding energy (eV)	concentration (%)
gSi_800°C_	Si–C–O/Si–O	102.61	96.50
	Si–C	101.20	3.50
gSi_950°C_	Si–C–O/Si–O	102.66	82
	Si–C	101.16	18

### 3.4 Mode-I Interlaminar Fracture Toughness Measurements

**Figure 5:**
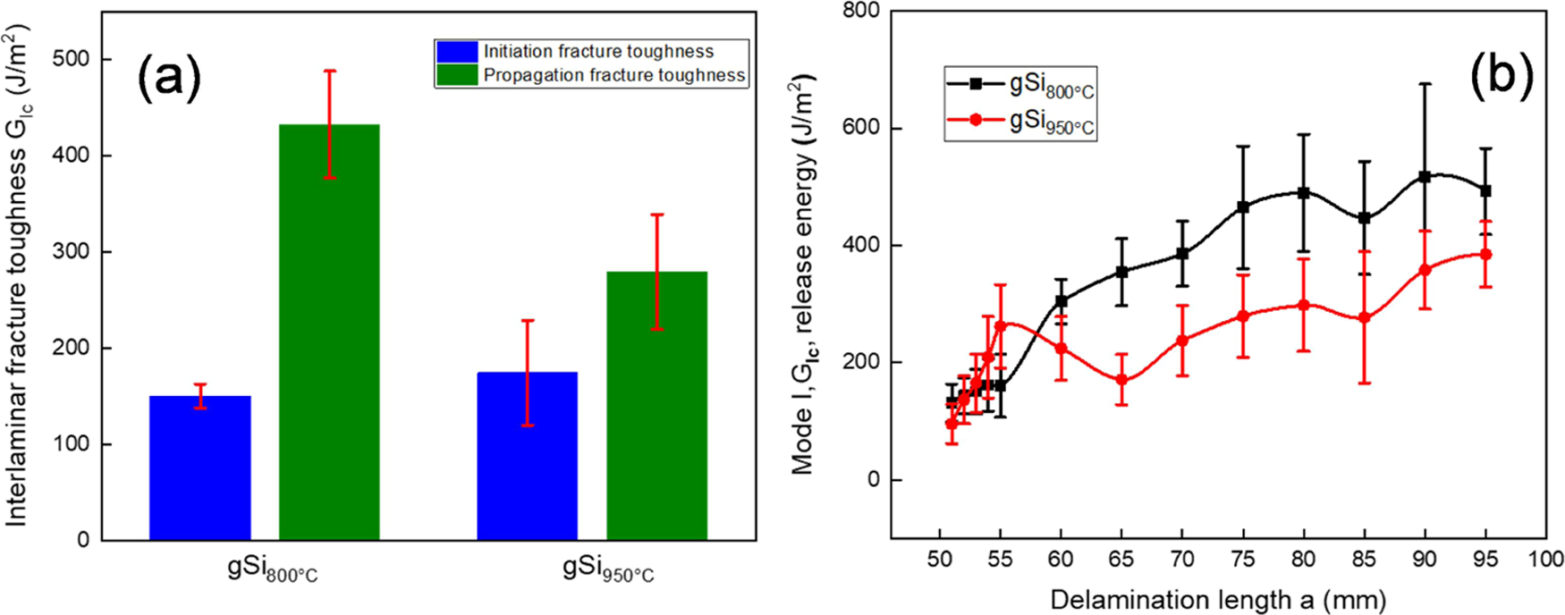
Mode-I interlaminar fracture toughness results of gSi_800°C_ and gSi_950°C_ samples. (a) Average values of initiation and propagation mode-I toughness. (b) *R*-curves of the tested specimen. Error bars represent standard deviation from five independent measurements.

Interlaminar fracture toughness, which was defined with regard to the mode-I strain energy release rate, was referred to as G_IC_ (Figure 5). During this test, delamination was revealed as a result of crack initiation and subsequently crack propagation at the laminate’s interlaminar area, triggered by a mixture of tensile and shear stresses. For this test, fracture toughness due to crack initiation (*G*_IC,INIT_) and crack propagation (*G*_IC,PROP_) was calculated (Table 5). Consequently, according to ASTM D5528,^42^ delamination *R*-curves were plotted utilizing the recorded load–displacement data (Figure 5b). A comparison with bCF and gCF mode-I interlaminar fracture toughness data and constructed *R*-curves reproduced by our previous work^29^ can be found in Supporting Information S4 (Figure S4). It can be seen that all GNF-based composites showed improved propagation and initiation *G*_IC_ values, when benchmarking them to the reference bCF sample (Supporting Information S4, Figure S4), with the only exception being gSi_950°C_, which exhibited a decrease of 23%. The best performance was presented by the gCF sample (Supporting Information S4, Figure S4), with 93.82 and 63.93% in initiation and propagation, respectively. In our previous study,^29^ we showed that a GNF interface can reinforce dramatically the interlaminar fracture toughness of CFRP owing to the GNFs’ capability to improve the interply adherence and resistance of the cracks’ initiation and propagation into the laminated structures.

**Table 5:** Mechanical Performance of All Samples

sample	mode-I INIT/PROP (Jm^–2^)	mode-II NPC/PC (Jm^–2^)	tensile strength (MPa)
bCF	226 ± 15/293 ± 27	1168 ± 57/1182 ± 20	519 ± 47
gCF	437 ± 25/480± 45	1674 ± 70/1692 ± 60	531 ± 40
gSi_800°C_	150 ± 12/432 ± 56	1920 ± 171/1940 ± 184	624 ± 39
gSi_950°C_	174 ± 54/279 ± 60	642 ± 132/781 ± 87	565 ± 15

**Figure 6:**
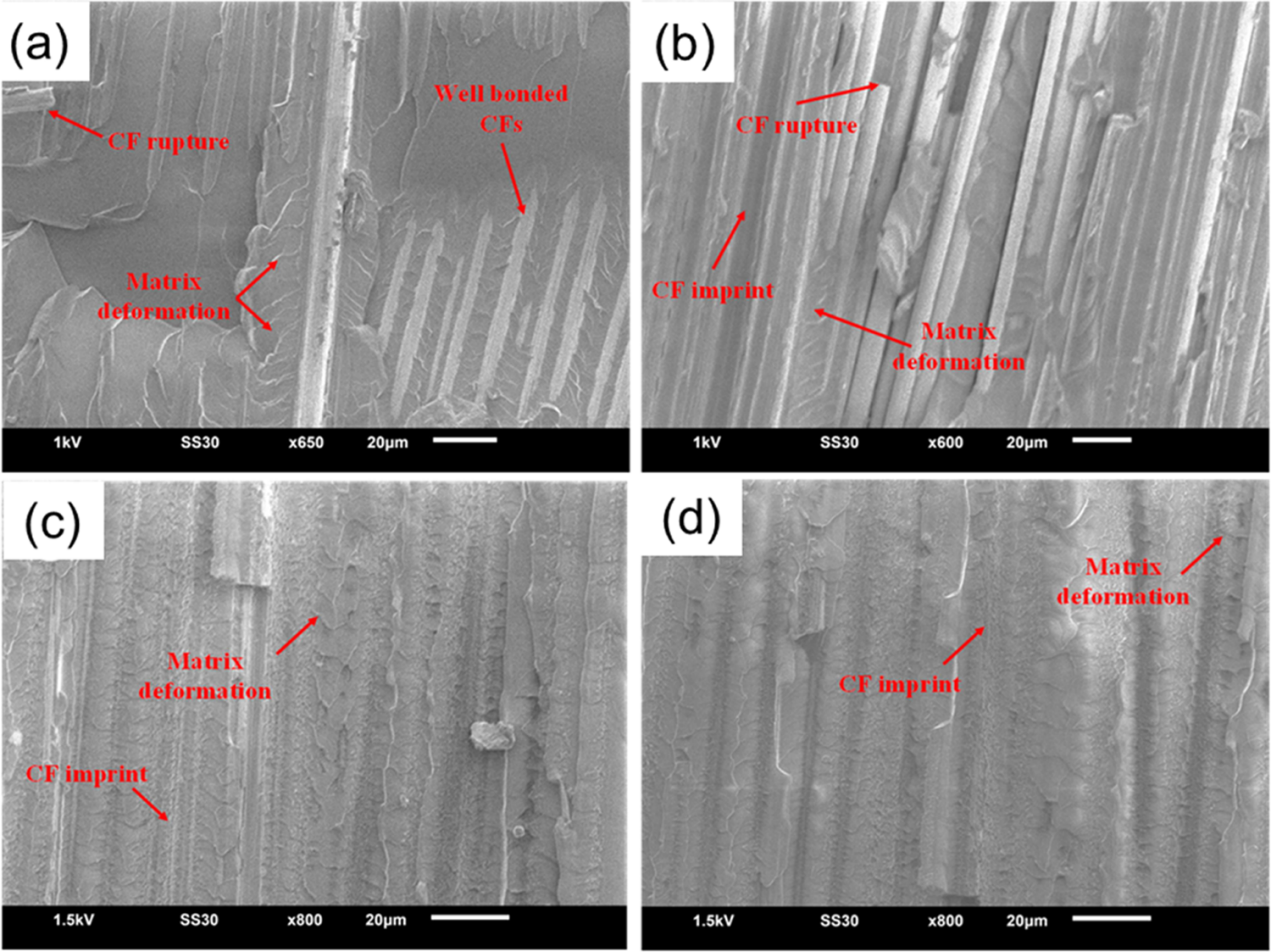
Fractographic micrographs of (a, b) gSi_800°C_ and (c, d) gSi_950°C_ samples.

Some fractographic micrographs were also acquired to investigate the failure mechanisms of the laminates during mode-I tests (Figures 6 and S5). A comparison with bCF and gCF data reproduced from our previous work^29^ can be found in the Supporting Information (Figure S5). From the images, it can be seen that for all coated samples (Figure 6a–d), there is matrix deformation after the test and a few CF imprints indicating CF pull-out failure mechanisms. However, not so many fibers were pulled out of the polymer matrix (better adhesion between the fibers and the matrix), and this is the reason why they present a tougher interface when compared to all other samples. The poor performance of gSi_950°C_ is attributed to the high temperature involved in the growth of the nanostructure.

### 3.5 Mode-II Interlaminar Fracture Toughness Measurements

**Figure 7:**
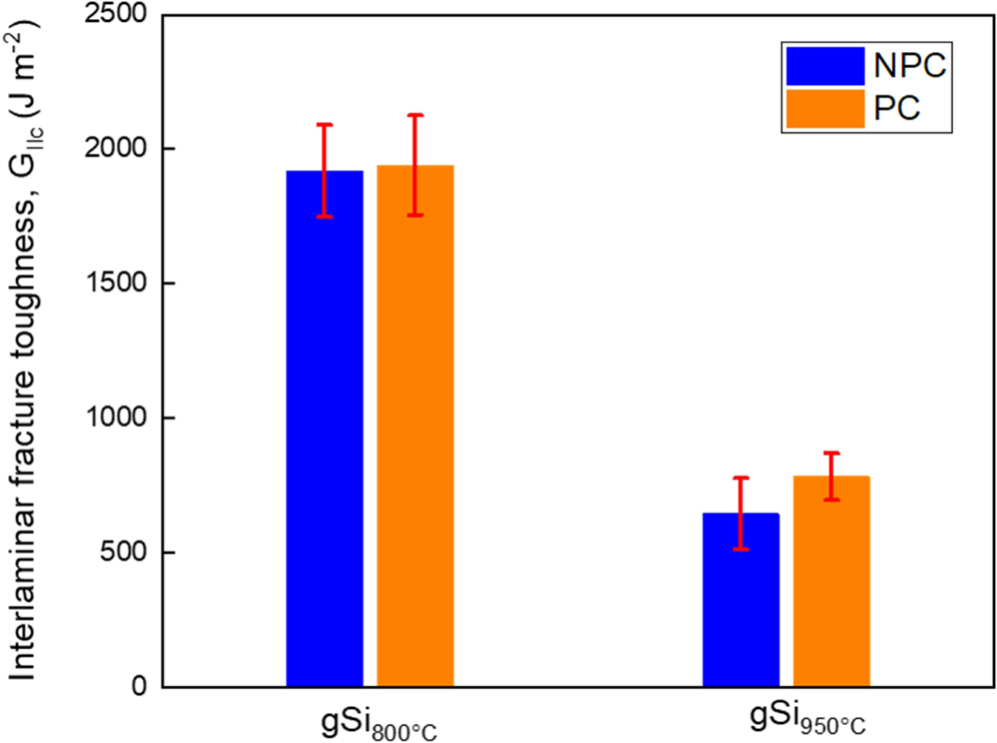
Mode-II interlaminar fracture toughness results for gSi_800°C_ and gSi_950°C_ samples. Error bars represent standard deviation from five independent measurements.

The fracture toughness was likewise assessed regarding the mode-II strain energy release rate (*G*_IIC_) (Figures 7 and S6). A comparison with bCF and gCF data reproduced from our previous work^29^ can be found in the Supporting Information (Figure S6). The outcomes of *G*_IIC_ did not follow a similar trend as the *G*_IC_ data, with gSi_800°C_ exhibiting the greatest performance of 64.34 and 64.04% improvements in NPC and PC tests, respectively (Figure 7 and Table 5). The second best sample (gCF, Supporting Information, Figure S6) exhibited 43.28 and 43.05% improvements again in NPC and PC tests, while gSi_950°C_ showed a decrease in *G*_IIC_ of 44.99 and 33.94%, respectively. The disagreement in the reinforcement among *G*_IC_ and *G*_IIC_ is mainly due to the sensitivity of Si-doped GNFs to interlaminar crack propagation prompted in mode-I loading. This can be attributed to the diverse types of stresses demonstrated throughout these two tests, where the crack growth is determined by tensile stress in mode-I tests and by shear stress in mode-II.^43^ It appears that the path of the offside microseparations participated crucially during the two tests. Generally, in *G*_IC_, lateral growth of microseparations could be observed, but there is no indication of them in *G*_IIC_ if the compressive stress is sufficiently strong.^44^

**Figure 8:**
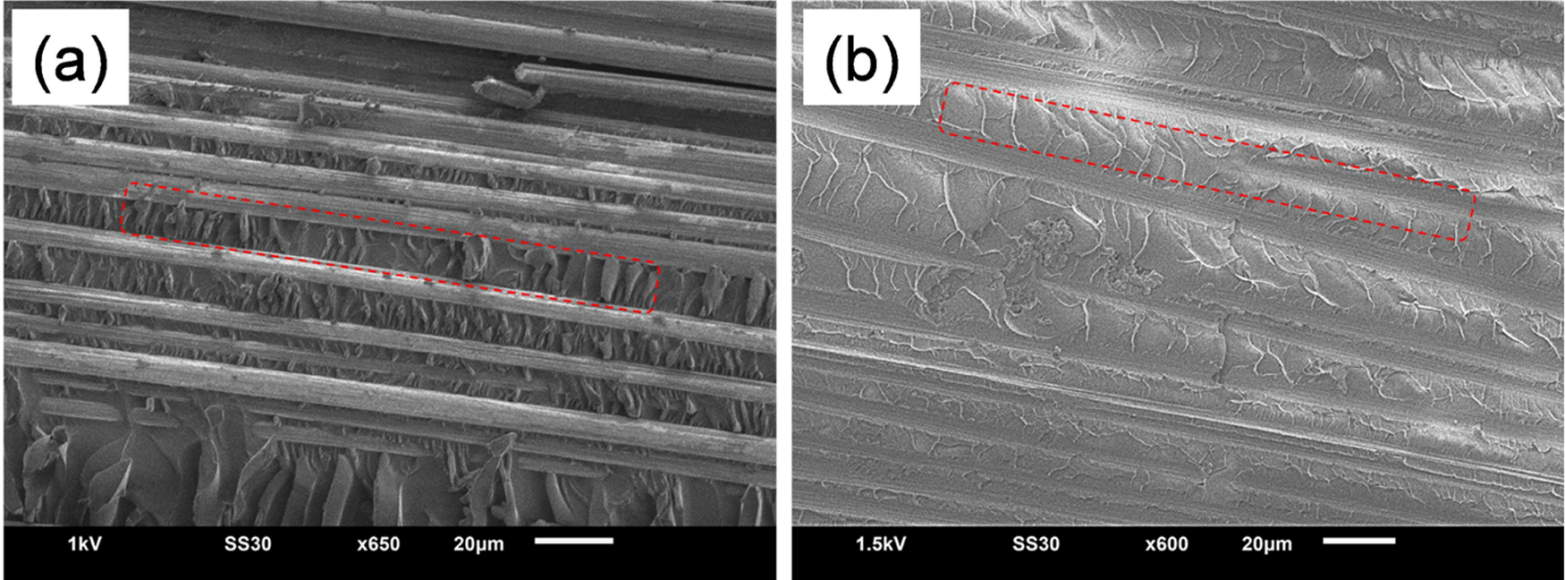
Postfailure fractographic analysis of mode-II tested samples. (a) gSi_800°C_ and (b) gSi_950°C_.

In order to understand better the failure mechanism in *G*_IIC_ testing, postfailure SEM images were obtained and analyzed. A comparison with bCF and gCF data reproduced from our previous work^29^ can be found in the Supporting Information (Figure S7). The postfailure images (Figures 8 and S7) showed that all samples except for the gSi_950°C_ sample exhibited a “rougher” surface, when benchmarking them to the reference bCF sample (Figure S7a). Observing the cracked surface of the composites, hackles (microcracks) could be identified, which constitute the main evidence of shear stresses’ manifestation during the interlaminar fracture test. Hackles initially form as microcracks, stimulated through tensile stresses on the interlaminar sheared area. While the hackles propagate, they arrive at the fiber layers and change their course owing to the presence of hybrid SiC/GNF heterostructures. The hackle motif is indicative of a shear stress governing state, not observed during mode-I. The bCF sample (Figure S7a) had several CF breaks and matrix deformation, with no obvious hackle motifs. gCF showed a moderate population of hackles (Figure S7b) with large lateral dimensions, which was in good agreement with the enhancement in *G*_IIC_. Instead, the hackles in gSi_800°C_ (Figure 8a) samples were ample and denser, making the results in mode-II PC tests by far the best ones. By analyzing the postfailure micrographs of gSi_800°C_, it is clear that shear stress stimulated microcracks having smaller sizes when compared to the other composites, but the densest motif between them. However, the hackles at gSi_950°C_ (Figure 8b) appeared smooth and not that dense, with not many CF ruptures, indicating a premature failure of the composite with not much resistance in the interlaminar region. There are a number of CF imprints as well, indicating again that they were completely pulled out from the epoxy without any particular resistance.

### 3.6 Tensile Strength Measurements

**Figure 9:**
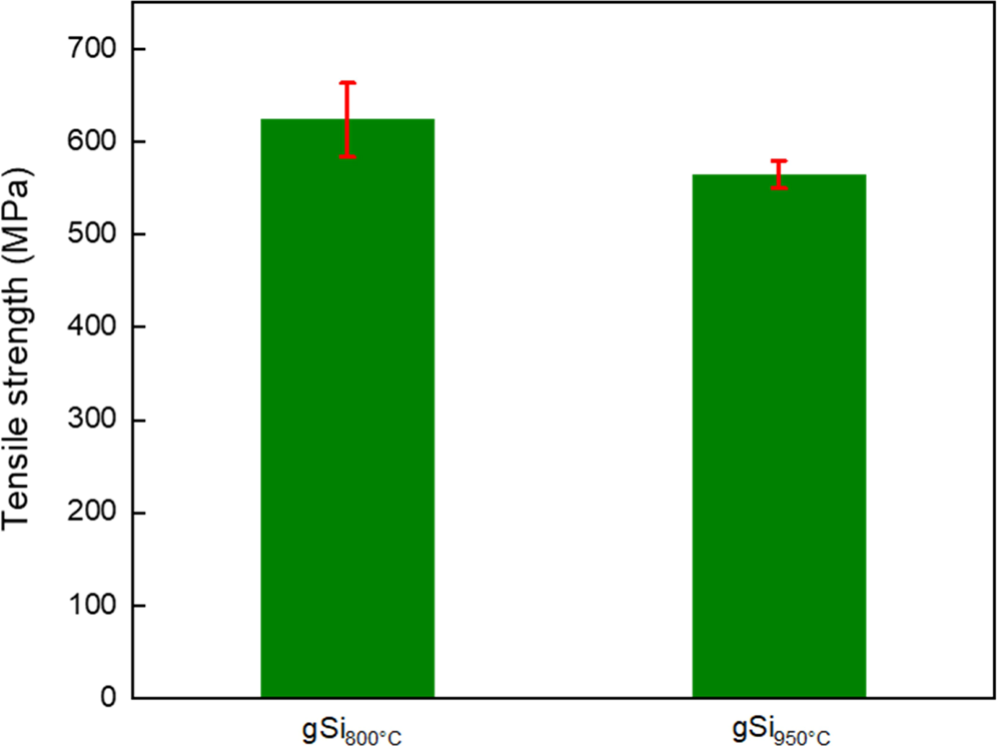
Tensile strength measurements of gSi_800°C_ and gSi_950°C_ samples. Error bars represent standard deviation from five independent measurements.

Tensile strength tests of the fabricated composites were performed to investigate the effect of directly grown SiC/GNFs on the tensile strength of samples (Figure 9). A comparison with bCF and gCF data reproduced from our previous work^29^ can be found in the Supporting Information (Figure S8). The configuration studied here incorporated two SiC/GNF-coated weaves positioned in the middle area of the laminate, opposing each other. However, the results presented for the gCF laminate were outsourced from our previous work,^29^ which have a total of four deposited layers, with two located in the middle and two layers at the outer surfaces. The gSi_800°C_ specimen exhibited the highest enhancement of about 20.18%, in contrast to gCF, which displayed an almost unchanged tensile strength, when compared to the reference specimen. The reinforcement of gSi_950°C_ was about 8.79%. The increased aspect ratio (height/lateral length) of the Si-doped GNFs in gSi_800°C_ and gSi_950°C_ samples provided a more effective stress transfer load when compared to the gCF sample. However, the crucial impact of thermal loading on the CFs could be seen clearly from the results of the gSi_950°C_ sample, where there was only a slight increment in the tensile strength when compared to gSi_800°C_.

### 3.7 Thermal Conductivity Measurements and Multifunctional Efficiency

**Figure 10:**
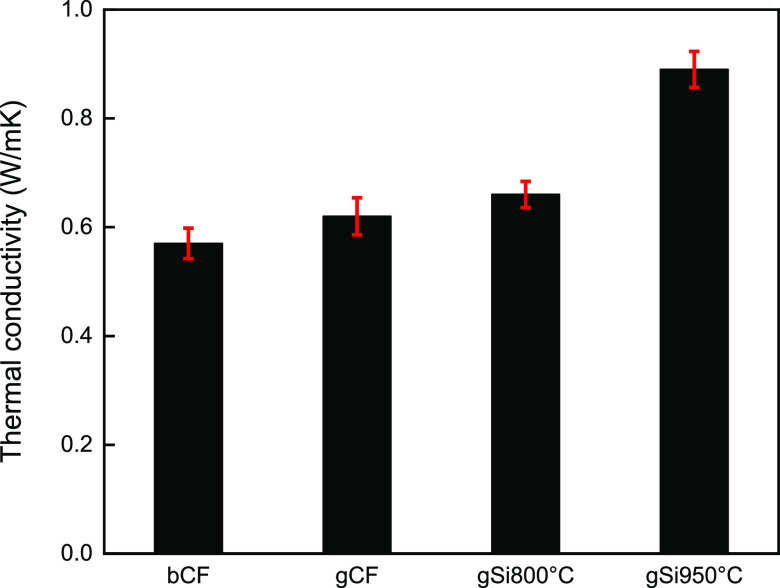
Thermal conductivities of bCF, gCF, gSi_800°C_, and gSi_950°C_ samples.

**Figure 11:**
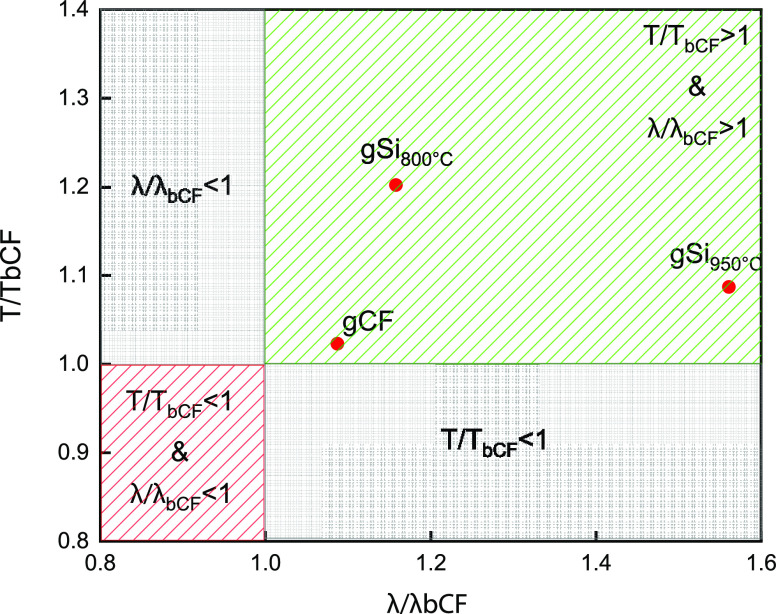
Multifunctional efficiency plot of bCF, gCF, gSi_800°C_, and gSi_950°C_ samples.

Herein, we introduce the concept of multifunctional efficiency, which is defined as the tensile strength normalized to the bCF specimen (*T*/*T*_bCF_) and graphed versus the TC of each sample (Figure 10) normalized to the bCF (λ/λ_bCF_), to assess the multifunctional performance of the laminated structures (Figure 11). Moreover, an explanation is offered on why the SiC/GNF framework exhibited increased TC when compared to the pure GNFs.

From the results, it can be seen that the SiC/GNF samples gSi_800°C_ and gSi_950°C_ exhibited increased TC when compared to the other samples, of about 15.79 and 56.14%, respectively. The gCF sample showed an increment of 8.77% when compared to the control bCF sample. This increment is attributed to the deposited conductive GNFs. However, further increment in the TC of the SiC/GNFs was attributed mainly to the SiC nanocrystals onto the GNFs, which are known for their exceptional TC.^45^

From the multifunctional efficiency plot (Figure 11), it can be seen that all fabricated samples are actually inside the desired area of multifunctionality (*T*/*T*_bCF_ > 1 and λ/λ_bCF_ > 1), making them suitable for applications, where excellent mechanical and thermal performance is needed.

**Figure 12:**
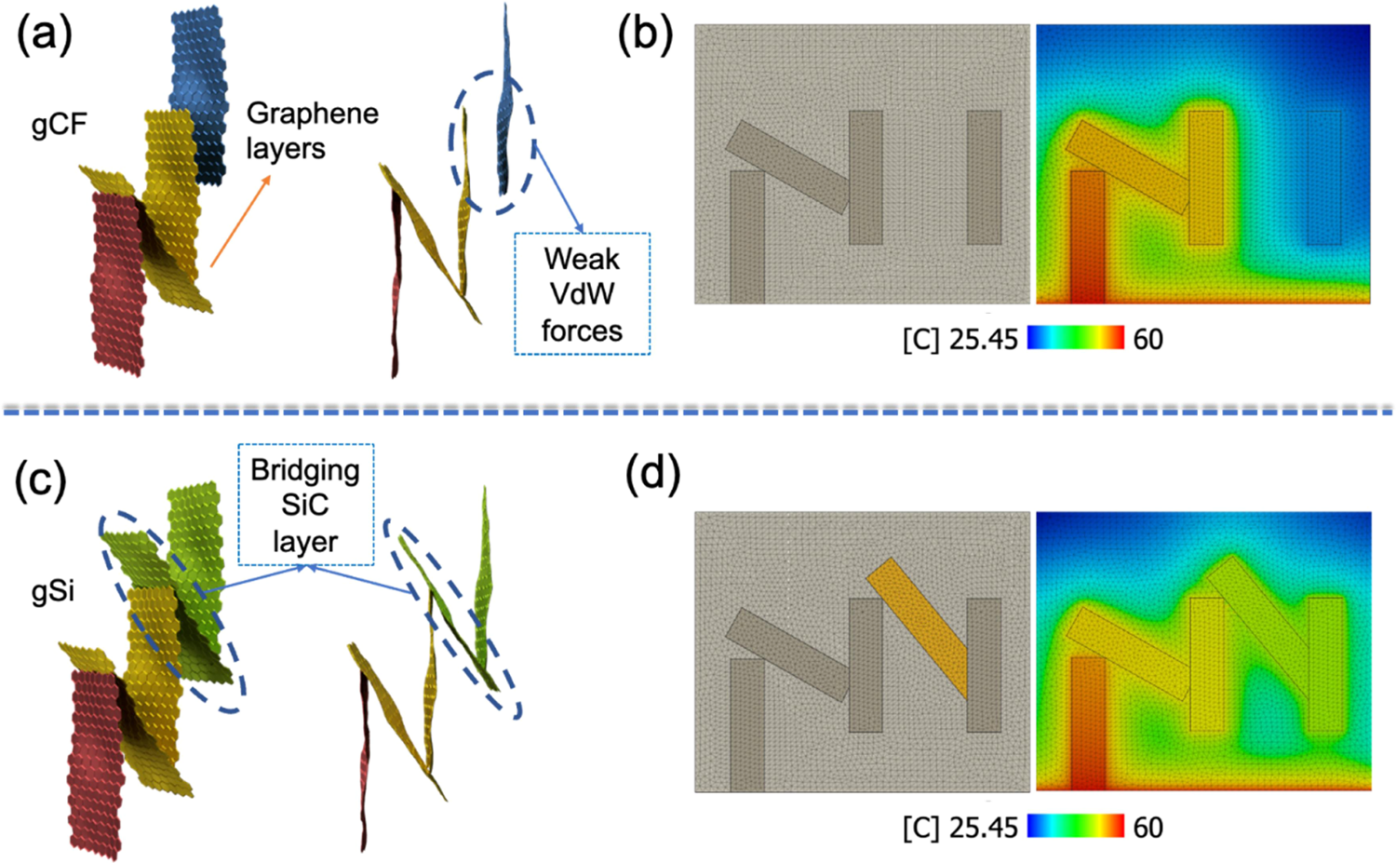
3D representation of the GNF and SiC/GNF heterostructures and the FEA model for heat dissipation into the CFRP composite. (a, b) gCF (only graphene layers) and (c, d) SiC/GNF interface. In figures (b, d), the dark-gray colored bars indicate the pure GNFs and the yellow ones indicate the SiC.

It has been established^19^ that the introduction of covalent bonding between two-dimensional (2D) layers can enhance the phonon transport when compared to van der Waals interaction. GNFs consist of a network of predominately vertically aligned graphene layers, which are coupled with van der Waals forces and are interconnected at junctions by covalent bonds. In SiC/GNFs, many graphene layers are interlinked covalently to SiC nanocrystals, providing longer continuous paths, thus increasing the mean free path of phonons, resulting in a higher TC along the out-of-plane vertical direction. Unlike GNF composites populated by weak interlayer van der Waals coupling, the through-thickness TC of SiC/GNF carbon fiber-reinforced epoxy composites is improved due to the much lower interfacial thermal resistance between graphene and covalently bonded SiC nanocrystals. Figure 12 depicts a simplistic model of GNF and SiC/GNF networks, along with a typical thermal model (Supporting Information S7, Table S1) for heat dissipation. Figure 12a illustrates the presence of both weak van der Waals interactions between graphene layers and covalent bonding at the intersections encountered in GNFs. The phonon transport is hindered when graphene layers are coupled by weak van der Waals forces, as a result, the TC is limited (Figure 12b), leaving the last graphene layer (blue color) at a lower temperature than the covalently bonded ones. In contrast, in Figure 12c (SiC/GNFs), the weak van der Waals interaction between the graphene layers was replaced by stronger covalent links with SiC. The benefit of this covalently bonded, continuous structure is the elimination of phonon scattering at the interface between adjoining sheets. The simulated heat map in Figure 12d shows that the covalent bridge between the SiC and graphene helped the phonon transport, as revealed by the display of a higher temperature between the SiC layer and the graphene layer, when compared to the pure graphene structure (Figure 12b).

## 4 Conclusions

In summary, a SiC/GNF heterostructure was developed by a one-step facile PECVD method using a TMS/CH_4_ mixture as a precursor. The SiC/GNFs show a characteristic structure composed of hierarchical graphene flakes decorated with a SiC nanocrystal architecture, leading to an enhanced through-thickness thermal conductivity by 56% compared to that of the pristine CFRP laminate. Some of the most important findings are as follows.

(i) By increasing the growth temperature, the SiC nanocrystals’ atomic percentage was increased (3.5% at 800 °C vs 18% at 950 °C).
(ii) Growth at 800 °C (gSi_800°C_) resulted in a heterostructure governed mostly by the GNFs and not that much from the SiC (3.5%). At 950 °C, the higher thermal loading along with the brittle nature of the populated SiC nanocrystals could induce a brittle interface to the interlaminar area of the fabricated composites, resulting in a decline in mechanical performance.
(iii) However, the thermal conductivity of gSi_950°C_ was enhanced tremendously by 56%, and this is attributed mainly to the increased quantity of SiC nanocrystals.
(iv) The tensile strength of all manufactured composites was preserved or even increased, making our specific piling sequence a very important factor for preserving their strength.

Finally, we would like to emphasize that our systematic experimental study supported by simulated modeling revealed the critical role of SiC in providing enhanced thermal conductivity in the through-thickness direction compared to pure GNFs/CF and bare CF laminates. The benefit of covalently bonded SiC nanolayers with graphene layers is that it provides a continuous path in the vertical direction, eliminating phonon scattering between graphene sheets. The XPS data showed a significant increment of the SiC content at gSi950 °C when compared to that at gSi800 °C, which is in agreement with the thermal conductivity measurements.

Meanwhile, the growth mechanism of SiC/GNF heterostructures and the effect of various deposition parameters on their growth are also worthy to be investigated on their own right; these investigations could stimulate future theoretical studies and promote the exploitation of their properties in various applications.

Our work demonstrates for the first time the exclusive potential of novel SiC/GNF heterostructures for attaining at the same time strong and thermally conductive CFRP, addressing many challenges for migrating toward MEA.

## Supporting Information Available

SEM; Raman; XRD data; modes I & II; tensile strength measurements; finite element analysis of heat dissipation into the CFRP matrix; SiC yield estimation; and void content (%) calculations (PDF)

## Notes

The following data are under a Creative Common Attribution 4.0 International (CC BY 4.0): Figures S1(a, b), (c, d), S2(bCF, gCF), S4 (bCF, gCF), S5(a, b), (c, d), S6(bCF, gCF), S7(a),(b), and S8(bCF, gCF). It is attributed to A.K., A.G., J.K., P.K.S., and P.P., and the original version can be found in reference (29). The authors indicate that no changes were made in the above data.
