# Supplementary material for: Improving the Through-Thickness Thermal Conductivity of Carbon Fiber/Epoxy Laminates by Direct Growth of SiC/Graphene Heterostructures on Carbon Fibers
Source: ACS Omega. 2023 Jun 29;8(27):24406–17. doi: 10.1021/acsomega.3c01951 (PMC10339322; doi:10.1021/acsomega.3c01951)
Supplement: Supplementary file 1 — ao3c01951_si_001.pdf [file ao3c01951_si_001.pdf]

Supporting information

Improving the Through-Thickness Thermal Conductivity of Carbon Fiber/Epoxy Laminates by Direct Growth of SiC/Graphene Heterostructures on Carbon Fibers

Anastasios Karakassides[1,3], Abhijit Ganguly[1], Constantinos E. Salmas[2], Preetam K. Sharma[1,4] and Pagona Papakonstantinou[1,]*

[1]School of Engineering, Ulster University, Belfast, BT15 1AP, Northern Ireland, UK

[2]Department of Materials Science & Engineering, University of Ioannina, 45110 Ioannina, Greece

Keywords: graphene nanoflakes (GNFs), silicon carbide (SiC), heterostructure, thermal conductivity, tensile strength, carbon fiber reinforced polymer (CFRP)

---

*Corresponding author, e-mail address: p.papakonstantinou@ulster.ac.uk
[3]Present address: Department of Applied Physics, School of Science, Aalto University, Espoo, FI-02150, Finland
[4]Present address: Department of Chemical Engineering, Loughborough University, Loughborough, LE11 3TU, UK

S1. SEM micrographs

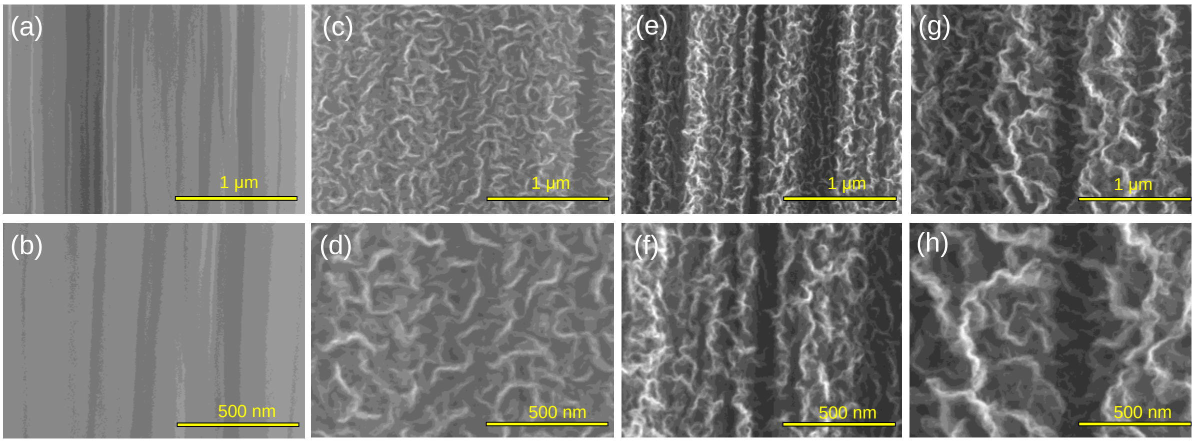

Figure S1. SEM micrographs of all samples for comparison purposes. (a-b) bare carbon fiber: bCF; (c-d) pure GNFs on CF: gCF; (e-f) SiC/GNFs on CF at 800 °C: $gSi_{800°C}$ and (g-h) SiC/GNFs on CF at 950 °C: $gSi_{950°C}$.

S2. Raman Analysis

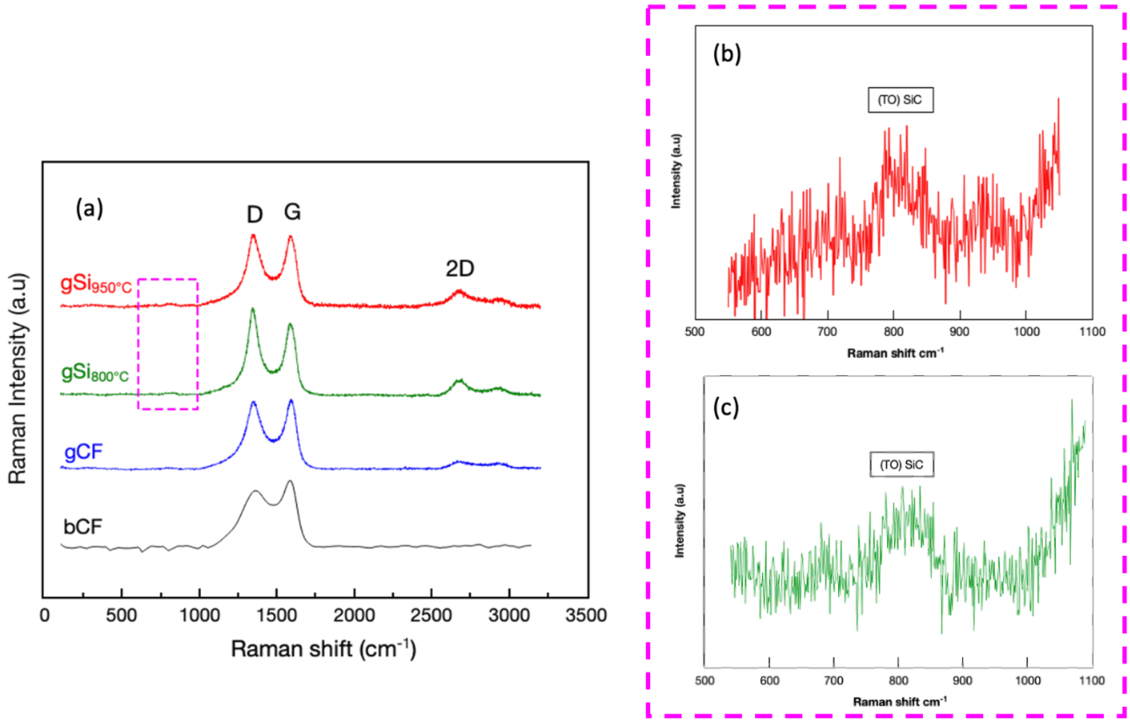

Figure S2. (a) Raman characterization of all samples. Bare CF: bCF; pure GNFs on CF: gCF; SiC/GNFs on CF at 800 ºC: $gSi_{800°C}$ and SiC/GNFs on CF at 950 ºC: $gSi_{950°C}$. (b) and (c) are Raman spectra of $gSi_{950°C}$ and $gSi_{800°C}$ respectively, of the dotted region indicated at (a),

S3. XRD measurements

The XRD spectra revealed only a carbon peak around 25.5 degrees with no differences observed between the two samples (same FWHM, position of the carbon band, intensity). The green highlighted area for both samples (increased intensity for $gSi_{950°C}$) is due to the copper tape used to mount the samples on the XRD sample holder.

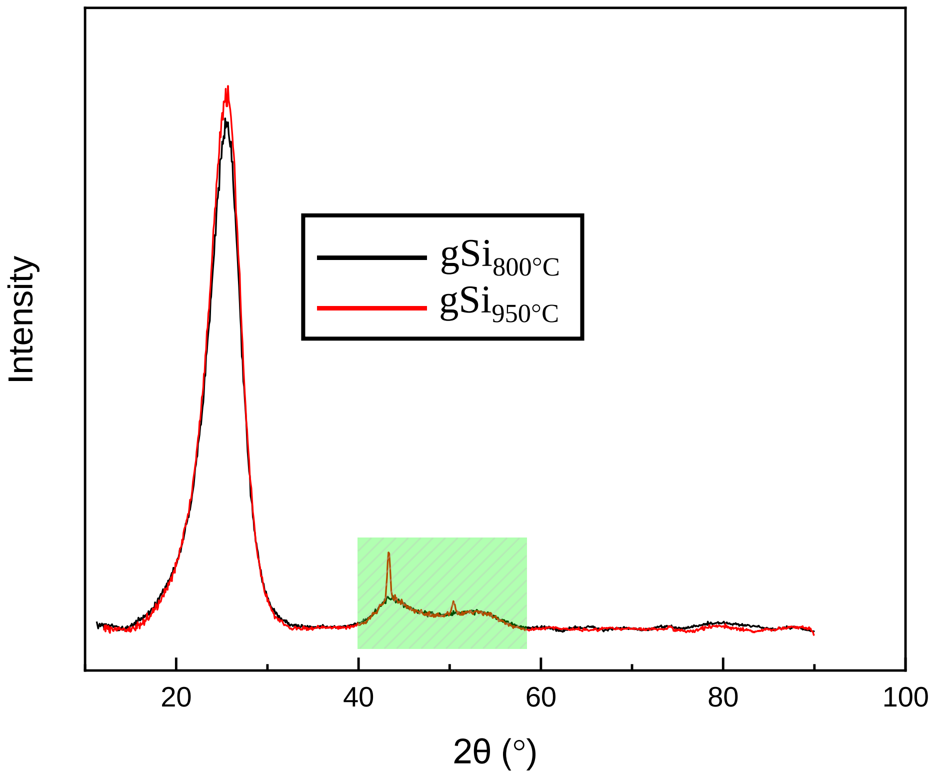

Figure S3. XRD spectra of $gSi_{800°C}$ and $gSi_{950°C}$ samples. $gSi_{800°C}$: SiC/GNF on CF at 800 ºC and gSi_{950}: SiC/GNF on CF at 950 ºC.

S4. Mode-I measurements

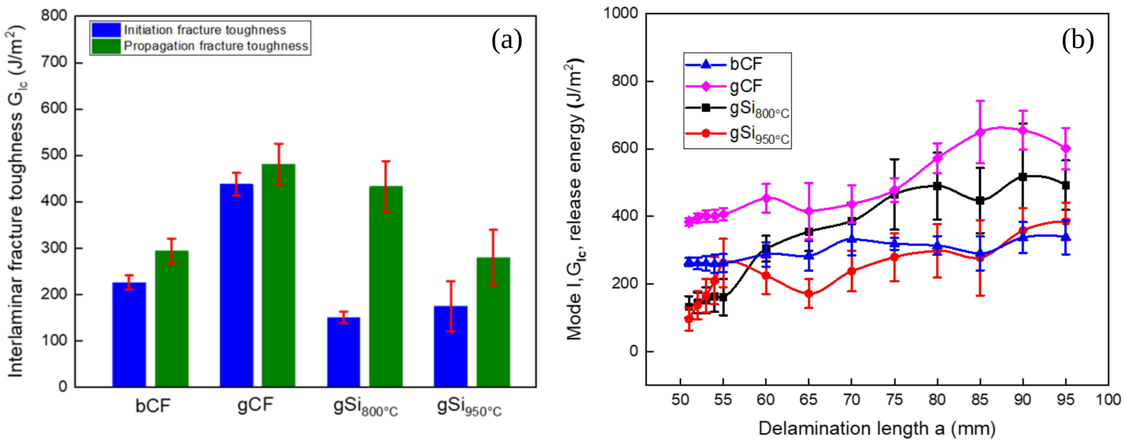

Figure S4. Mode-I interlaminar fracture toughness results for bCF, gCF, $gSi_{800°C}$ and $gSi_{950°C}$ samples. (a) Average values of initiation and propagation mode-I toughness, (b) R curves of all tested specimen. Error bars represent standard deviation from 5 independent measurements.

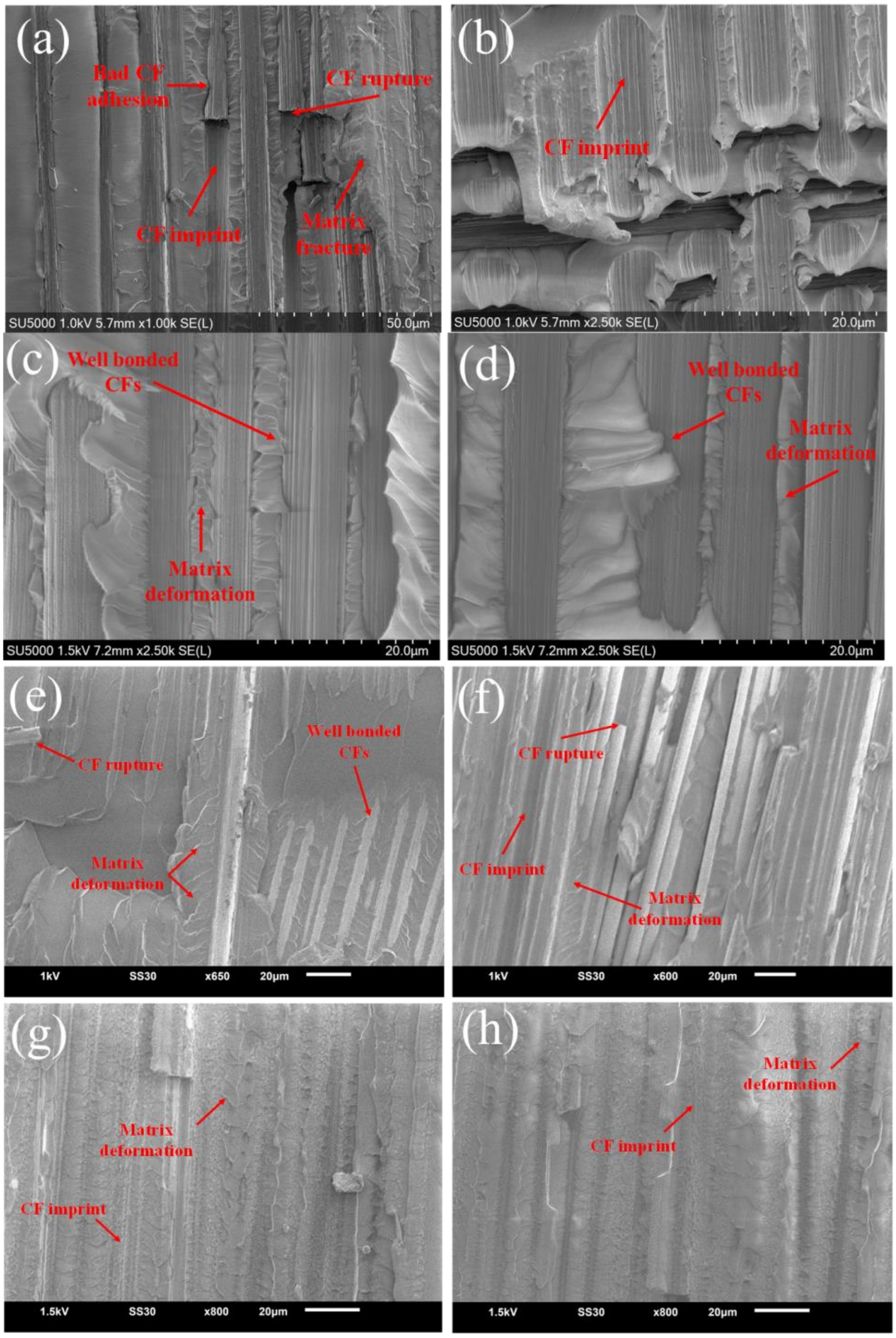

Figure S5. Fractographic micrographs of (a-b) bCF, (c-d) gCF, (e-f) $gSi_{800°C}$ and (g-h) $gSi_{950°C}$ samples.

S5. Mode-II measurements

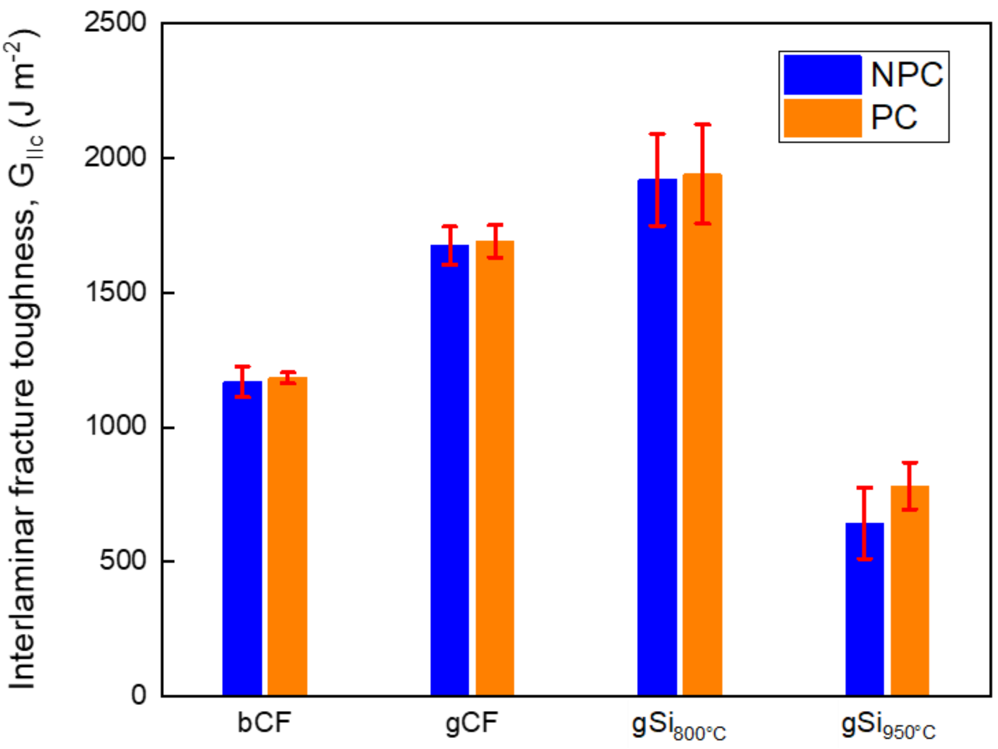

Figure S6. Mode-II interlaminar fracture toughness results for bCF, gCF, $gSi_{800°C}$ and $gSi_{950°C}$ samples. Error bars represent standard deviation from 5 independent measurements.

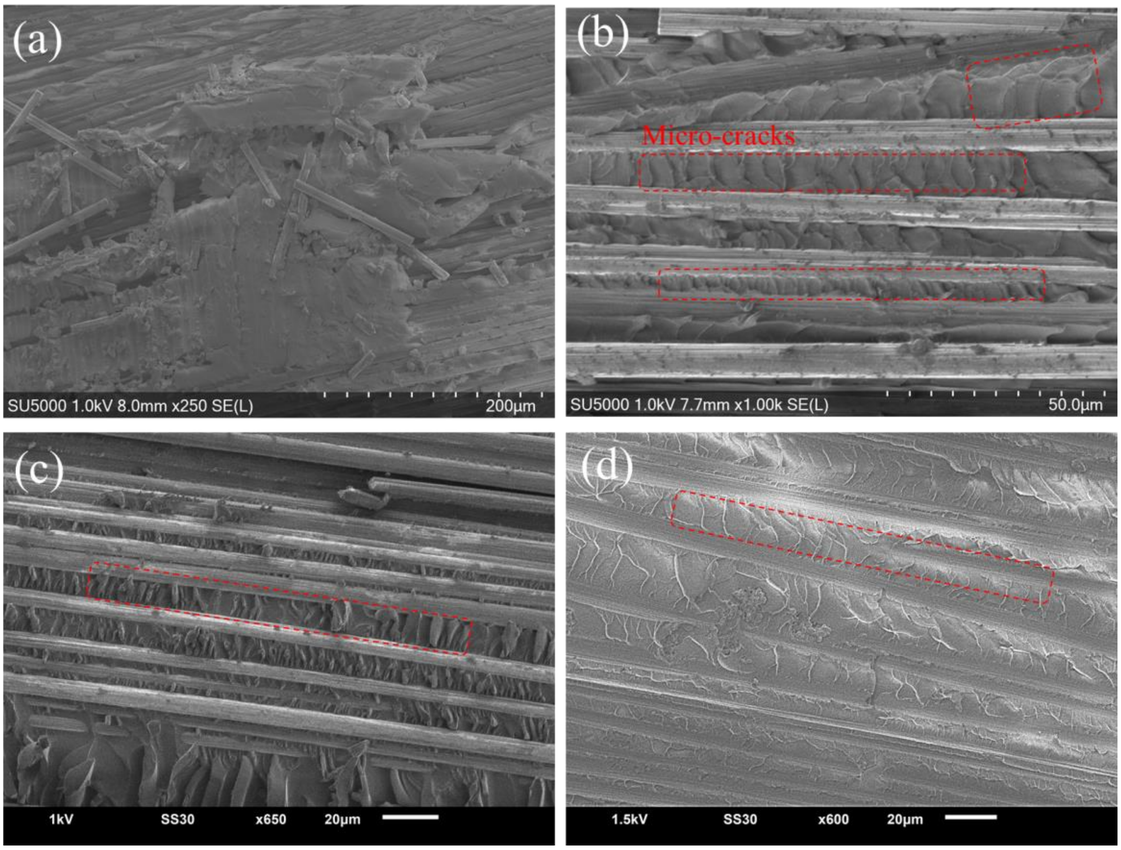

Figure S7. Post-failure fractographic analysis of mode-II tested samples. (a) bCF. (b) gCF, (c) gSi800°C and (d) gSi950°C.

S6. Tensile strength measurements

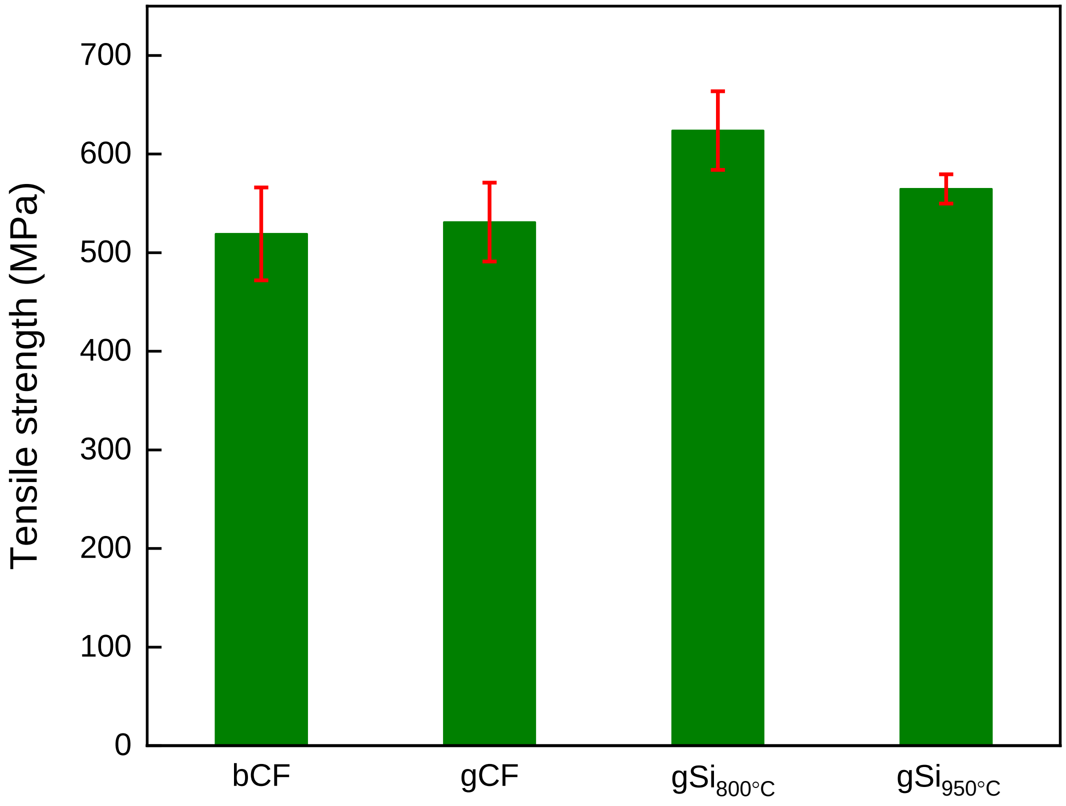

Figure S8. Tensile strength measurements of bCF, gCF, gSi800°C and gSi950°C samples. Error bars represent standard deviation from 5 independent measurements.

S7. Finite element analysis (FEA) of the heat dissipation into the CFRP matrix

Finite element analysis (FEA) software (Autodesk Fusion 360) was used to compare the heat dissipation of GNFs and the SiC/GNFs structures into the CFRP matrix. In a typical simulation (Figure 12), a simple thermal model under steady state conditions was selected in the software, where a temperature of 60 °C was applied linearly at the bottom face of the simulation matrix. The parameters used in the simulation are being quoted in the following table:

Table S1. Simulation parameters for the thermal model

**Sample**	**Convection Coefficient**	**Applied Temperature**	**Thermal Conductivity of GNFs**	**Thermal Conductivity of SiC**	**Thermal Conductivity of CFRP**

gCF	10 W m^{-2} K^{-}	60°C	680 W m^{-1} K^{-1}	-	0.56 W m^{-1} K^{-1}
gSi	10 W m^{-2} K^{-}	60°C	680 W m^{-1} K^{-1}	480 W m^{-1} K^{-1}	0.56 W m^{-1} K^{-1}

S8. SiC yield estimation

The SiC yield for our PECVD reactor can be defined by the following expression:

$$\text{Yield} = \frac{\text{area of deposition (cm}^2\text{) * (\% SiC/100) (XPS Si2p)}}{\text{collection time (min) *total gas flow (sccm)}} \quad [\text{cm}^2/\text{L}] \qquad \text{(S1)}$$

We used 10 sccm (0.01 L/min) total gas flow in all fabricated samples (8 sccm TMS + 2 sccm CH_4), 30 min total deposition time and the same deposition area. This practically means that by changing the % of SiC (we define as % of SiC, the % of Si-C bonding estimated from the XPS Si2p analysis (reported in **Table 4)** multiplied by the total amount of Si-(reported in **Table 3**) in every sample, we can roughly define the yield of the PECVD reactor and investigate the effect of different temperatures:

$$\text{Yield}_{\text{gSi800°C}} = \frac{21\text{ cm} \times 2\text{ cm * (0.06/100)}}{30\text{*}0.01} = 0.085\text{ cm}^2/\text{L}$$

$$\text{Yield}_{\text{gSi950°C}} = \frac{21\text{ cm} \times 2\text{ cm * (3.91/100)}}{30\text{*}0.01} = 5.5\text{ cm}^2/\text{L}$$

Hence, when comparing the two yields we can clearly see that:

$\text{Yield}_{\text{gSi950°C}}$ / $\text{Yield}_{\text{gSi800°C}}$ = 5.5 / 0.085 ≈ 65

That means that by increasing the temperature from 850°C to 950°C there is an enormous increase in the yield of SiC growth by 65 times.

S9. Void content (%) calculations

We used some cross section SEM micrographs to estimate the void content (%) of the bare CF composite. Because all samples were fabricated together using the VARI method we assume that the void contents are similar in all samples. The calculations were based on optical observations of the voids and more specifically on the %Area of the highlighted (red colour) voids, in comparison with the total %Area of every micrograph (units are pixels). With the help

of ImageJ, it was possible to obtain the %Area of the highlighted voids, which however is overestimating the real void content, because there are some other areas on the micrograph which are not actually real voids, but matrix deformation for example. However, we consider them all in our estimation.

According to these calculations the average %Area of the highlighted areas, is about 2.485%. So, we assume that the void content based on this area recognition procedure is <2.485% if we consider the overestimation discussed previously.

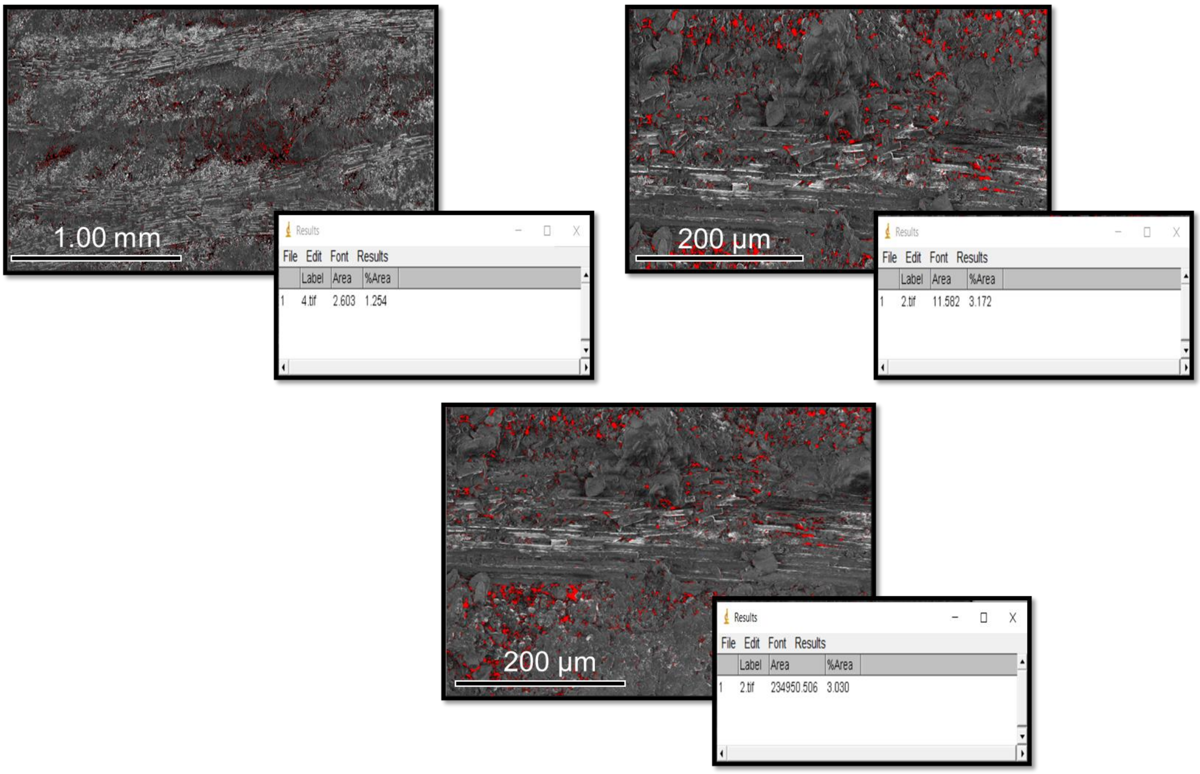

Figure S9. Image recognition of the voids using ImageJ.
